# Intra- and Inter-expert Validation of an Automatic Segmentation Method for Fluid Regions Associated with Central Serous Chorioretinopathy in OCT Images

**DOI:** 10.1007/s10278-023-00926-6

**Published:** 2024-01-12

**Authors:** Mateo Gende, Lúa Castelo, Joaquim de Moura, Jorge Novo, Marcos Ortega

**Affiliations:** 1 Grupo, VARPA, Instituto de Investigación Biomédica de A Coruña (INIBIC), Universidade da Coruña, Xubias de Arriba, 84, 15006 A Coruña, Spain; 2 Centro de investigación, CITIC, Universidade da Coruña, Campus de Elviña s/n, 15071 A Coruña, Spain

**Keywords:** Central serous chorioretinopathy, Deep learning, Segmentation, Computer-aided diagnosis, Ophthalmology

## Abstract

Central Serous Chorioretinopathy (CSC) is a retinal disorder caused by the accumulation of fluid, resulting in vision distortion. The diagnosis of this disease is typically performed through Optical Coherence Tomography (OCT) imaging, which displays any fluid buildup between the retinal layers. Currently, these fluid regions are manually detected by visual inspection a time-consuming and subjective process that can be prone to errors. A series of six deep learning-based automatic segmentation architectural configurations of different levels of complexity were trained and compared in order to determine the best model intended for the automatic segmentation of CSC-related lesions in OCT images. The best performing models were then evaluated in an external validation study. Furthermore, an intra- and inter-expert analysis was conducted in order to compare the manual segmentation performed by expert ophthalmologists with the automatic segmentation provided by the models. Test results of the best performing configuration achieved a mean Dice of $0.868 \mathop {\pm } 0.056$ in the internal dataset. In the external validation set, these models achieved a level of agreement with human experts of up to 0.960 in terms of Kappa coefficient, contrasting with a value of 0.951 for agreement between human experts. Overall, the models reached a better agreement with either of the human experts than these experts with each other, suggesting that automatic segmentation models for the detection of CSC-related lesions in OCT imaging can be useful tools for assessing this disease, reducing the workload of manual inspection and leading to a more robust and objective diagnosis method.

## Introduction

Central Serous Chorioretinopathy (CSC) is a retinal disease that causes visual impairment characterised by the detachment of the retina due to the accumulation of Subretinal Fluid (SRF) produced by the dysfunction of the retinal pigment epithelium and the hyperpermeability and enlargement of the underlying choroid. Patients with CSC typically experience central vision loss, central scotoma, micropsia or metamorphopsia [1]. This disease was first described in 1866 by Albrecht von Graefe as central recurrent retinitis and involved the detachment of the serous retina, primarily affecting the macular region [2].

Recent research has shed some light on the causes of CSC, pointing towards choroidal vascular hyperpermeability, which can lead to an increase in the hydrostatic pressure beneath the retinal pigment epithelium (RPE), causing it to disintegrate [3]. The balance between oncotic and hydrostatic pressure at the RPE normally results in fluid flowing from the retina into the choroid. However, in CSC, the increase in hydrostatic pressure within the choroid causes fluid to accumulate beneath the RPE. When the hydrostatic pressure beneath the RPE is high, it pushes the RPE forward, causing a discontinuity in its barrier, leading to the detachment of the RPE and punctate areas of leakage, commonly referred to as “microrips” or “blowouts”.

The presence of SRF leakage and accumulation can cause a loss of vision, as seen in the study conducted by Mrejen et al. [4], which researched the long-term causes of vision loss in the CSC. Different medical imaging techniques have been put forward for the diagnosis of this disease including, but not limited, to fluorescein angiography, indocyanine angiography, fundus autofluorescence imaging, Colour Fundus Photography (CFP) and Optical Coherence Tomography (OCT). With the former two being more invasive methods, the latter three are generally favoured due to their non-invasiveness, their lower risk of complication and convenience [3, 5]. These methods allow for a safe and effective monitoring of the disease and an early detection of vision loss.

In particular, OCT is a non-invasive imaging technique that can produce micrometre-resolution cross-sectional and volumetric visualisations of the retinal tissue. Its cross-sectional nature makes it the preferred imaging modality for the diagnosis of the CSC [6] as it enables the visualisation of different layers or sections of the eye [7, 8]. This, in turn, allows the visual inspection of the changes caused by disease progression [9, 10]. Its ability to allow the direct observation of the various layers that make up the macula makes it one of the most widely used techniques for the diagnosis of various ocular pathologies, including age-related macular degeneration [11], diabetic macular oedema [12], cystoid macular oedema [13], as well as CSC [14].

OCT enables an easy visualisation of any alterations related to CSC such as neurosensory detachment, detachment of the pigmentary epithelium, protrusion of the RPE, thickness changes in the posterior retinal surface, granulations on the detached retina, hyperreflective spots, RPE defects, RPE proliferation and subretinal fibrous exudates [10]. One of the most noticeable changes of CSC is the accumulation of SRF, which can appear in OCT imaging as a dark area around the pigmentary epithelium [15]. Early detection of the CSC is crucial for avoiding serious symptoms such as vision loss. However, the diagnosis process of CSC through OCT is slow and time-consuming, as well as subjective and prone to errors or misdiagnosis as it is carried out manually by expert examiners [16]. In this situation, the use of deep learning models for the automatic segmentation in CSC diagnosis could greatly benefit the process and aid the experts in the assessment of this disease.

Deep learning models make use of several consecutive convolutional layers to identify patterns and structures in large datasets [17, 18]. These models are able to automatically learn patterns by means of annotated examples, without the need to formalise the explicit knowledge needed to perform certain tasks. This makes deep learning models especially attractive for fields such as medicine, where the ability of these models to automatically learn how to identify patterns of disease makes them invaluable in the development of new and advanced Computer-aided Diagnosis (CAD) systems.

The use of CAD systems to automate the diagnosis process of CSC can lead to increased efficiency and accuracy, reducing the risk of errors derived from subjective expert assessment. Given the relevance of this topic, several studies have employed fundus imaging for the diagnosis of CSC, such as in the work of Chen et al. [19], where a deep learning model was proposed for the automatic detection of CSC leak points. For this purpose, they employed an attention-gated network architecture, integrating an attention gate with convolutional layers. The results highlighted the performance of deep learning models for the detection of CSC leakage points. However, this study is limited by its reliance on fluorescein angiography imaging, an invasive procedure which requires the use of a contrast die to highlight the blood vessels. Xu et al. [20] developed a deep learning-based architecture for the screening of SRF from CFP images. The network architecture followed a cascade approach in which two separate Convolutional Neural Network (CNN) models are able to determine the presence or absence of the disease, and whether it affects the central foveal region. However, it does not provide a true segmentation map of the presence of fluid. More recently, Yoo et al. [21] used a different approach, training conditional generative models to create the segmentation maps of the area of interest in the lesions with presence of SRF, with results that approach those of the human annotation. In spite of this, the use of generative models for generating segmentation masks has a high risk of generating maps that may look convincing enough to fool the discriminator but have no bearing on the presence of SRF, as the results show. These studies indicate that fundus imaging can be used for the automatic characterisation of the CSC, and highlight the utility of deep learning architectures in extracting the relevant characteristics in this disease, but may require invasive procedures or are otherwise limited in the accuracy of the segmentation outputs they are able to provide.

On the other hand, the advantages of OCT have made this imaging technique increasingly popular for the diagnosis of retinal diseases, particularly for the detection of pathological fluid regions. The high-resolution, cross-sectional images captured by OCT, combined with the use of automatic segmentation techniques, can offer a comprehensive analysis of the affected area, which can be crucial for a precise diagnosis. Previous works have shown the potential of deep learning-based segmentation in OCT images for various retinal diseases, such as serous retinal detachment [22], diabetic macular oedema [23], glaucoma [24], age-related macular degeneration [25] and intra-retinal cysts [26].

Because of this, recent studies have employed OCT to automatically analyse the CSC. Gao et al. [27] presented a study in which they employed an area-constraint fully convolutional network to perform the automatic segmentation of the CSC region in OCT images. The results showed that the model was close to manual segmentation after independent layer segmentation as well as quantitative and qualitative evaluations. However, this methodology was trained and tested only on a small dataset consisting of 10 eyes, 5 of which suffered CSC. Rao et al. [28] conducted a study to automatically segment regions affected by CSC in OCT images using deep learning-based architectures. This methodology relied on a pre-processing stage in order to adapt the images to the architecture, which was trained and validated on a similarly small dataset of only 15 eyes annotated only by a single expert. In the work of de Moura et al. [29], the authors proposed an end-to-end methodology for the automatic identification and segmentation of intra-retinal fluid regions associated with CSC in OCT scans. To achieve this, the authors adapted a fully convolutional architecture inspired by the SegNet architecture [30], while omitting any pre- or post-processing stages. This approach was validated on a larger dataset than the two other approaches. However, the images were only annotated by a single expert, which poses a risk of biasing the results towards that single expert. Pawan et al. introduced a modification to capsule networks based on dilation, residual connections, inception blocks and capsule pooling in order to better adapt the architecture to the segmentation of fluid in images of CSC patients. These changes also reduced the overall complexity of the networks while maintaining competitive performance. Nevertheless, its evaluation is based on the annotations of a single expert, which may pose risks of bias, similarly to the other previous approaches. Indeed, these studies highlight the recent efforts dedicated to the automatic analysis of the CSC in OCT imaging.

Recent works on the automatic segmentation of pathological and CSC-related lesions using OCT have shown promising results, while providing a clear and accurate visualisation of the progression of the disease. Nevertheless, these results are based on the annotations of a single expert which, as previously mentioned, can lead to subjectivity. Moreover, these studies may not fully capture the nuances of the manual diagnosis process. In order to address this limitation, an intra- and inter-expert analysis is necessary, so that the variability and subjectivity typically associated with manual inspection can be properly assessed. This way, a more robust and reliable assessment of the diagnosis can be provided, by taking into account any inconsistencies and potential disagreements among experts.

In this work, we aim to address this crucial challenge by presenting a comprehensive study in the application of deep learning models to the automatic segmentation of SRF regions in OCT images associated with CSC. Complementarily, this study is extended by including an intra- and inter-expert analysis of the best performing models with multiple expert ophthalmologists. These models were trained and validated using a representative dataset of the pathology (specifically designed for this study), along with an external validation dataset used for the intra- and inter-expert analysis. This analysis can provide ground-breaking evaluation of any possible inconsistencies among the automatic segmentation models as well as valuable insight into inter-expert disagreement. The main contributions of this work can be summarised as follows:

- This work presents a comparative analysis of various modular deep learning architectural configurations for fluid segmentation.
- This analysis is complemented by an evaluation of the configurations that produced the best results in an external dataset annotated by two human experts.
- The intra- and inter-expert analysis that was performed revealed that the deep learning models exhibited better alignment with individual human experts, surpassing human inter-expert consistency.
- Intra- and inter-expert analysis can set a new standard for the validation of future studies in the field.

## Materials and Methods

**Fig. 1:**
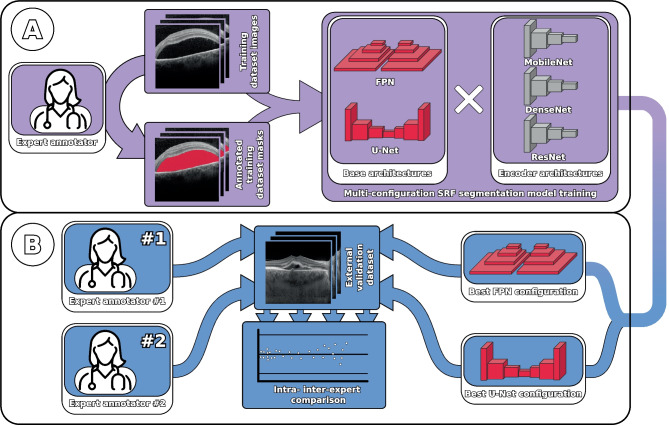
Summary of the experiments that were performed. **A**: Multi-configuration SRF segmentation model training. Six representative configurations of backbone segmentation network and encoder architectures were trained and validated on an OCT image dataset. **B**: Intra- and inter-expert comparison. The best model configurations for each backbone architecture were selected and compared among themselves and with two expert annotators on an external validation dataset

In this work, we propose a deep learning-based methodology for the automatic segmentation of fluid regions in OCT images of patients with chronic CSC. The methodology is comprised of two main stages, as displayed in Fig. 1. The first stage involves the training and validation of a series of deep learning models using a representative dataset of OCT images belonging to CSC and healthy control patients. The second stage involves an intra- and inter-expert analysis between the best performing models of the first stage and multiple expert ophthalmologists. Using an external validation dataset, the automatic segmentations produced by the models are compared among themselves and with the manual annotations produced by the human experts. This comparison provides a thorough and comprehensive analysis of the performance of the automatic models, and can offer valuable insight into inter-expert disagreement.

### Automatic Segmentation of Fluid Regions

The first stage of the methodology is focused on training, evaluating and comparing the performance of several prominent deep learning architectures for the segmentation of the fluid regions in OCT scans of patients with CSC.

#### Dataset

**Fig. 2:**
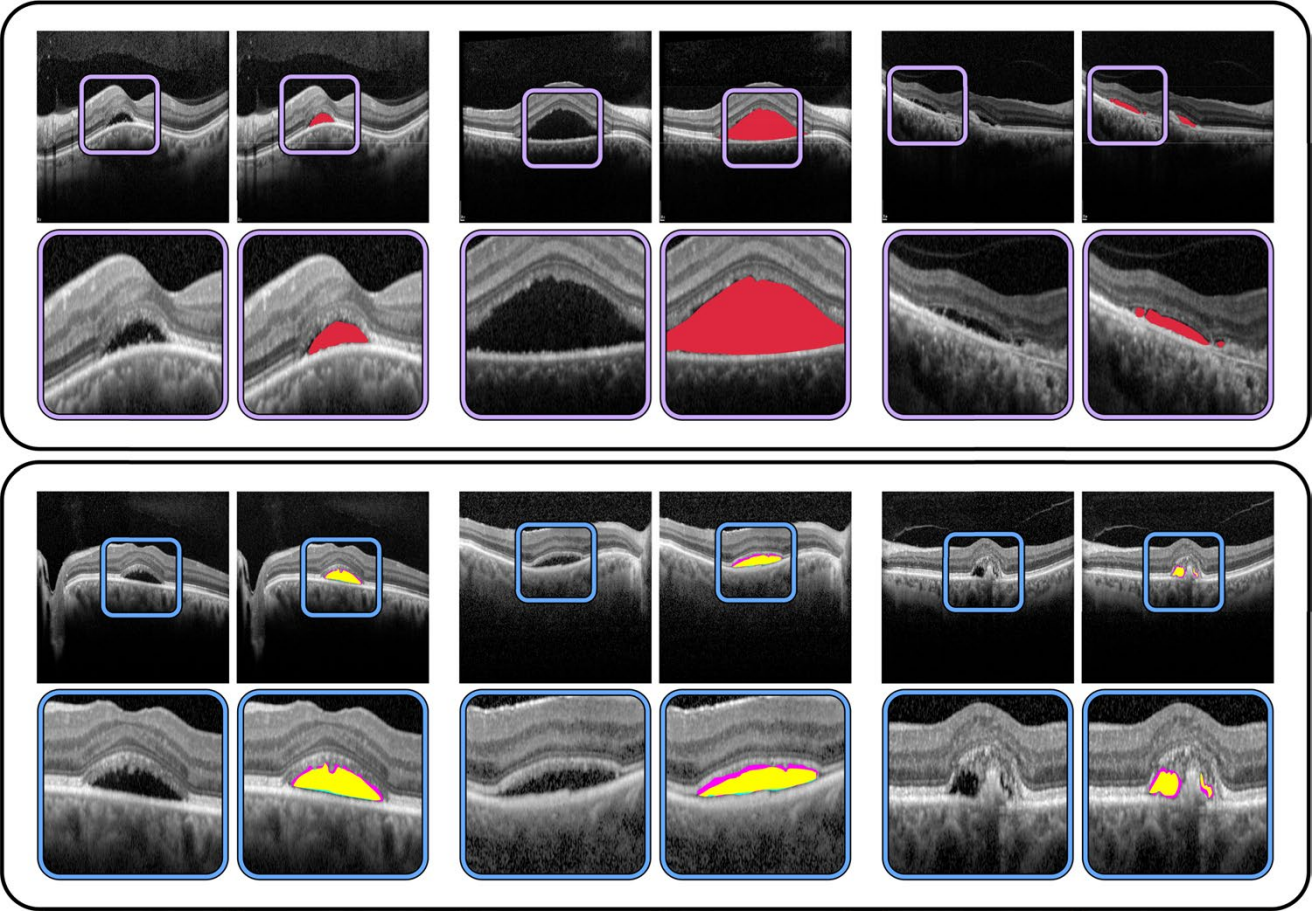
Representative examples from both datasets, along with a detailed view of the expert annotations. *Top*: First dataset, employed for training and validation of the models, expert annotations shown in red. *Bottom*: Second dataset, employed for the intra- and inter-expert analysis. In cyan, annotation from the first expert. In magenta, annotation from the second expert. In yellow, overlap between the two experts

A dataset was collected with a total of 557 OCT images from different patients. 303 images correspond to patients of CSC, while 254 display healthy control patients. These images were acquired with a Heidelberg spectralis^®^ optical imaging platform. These OCT scans are all macula centred, and were extracted from both the left and right eyes using different scanning protocols, including 1- and 7-line scans, the two highest quality scanning protocols most widely used in the assessment of the CSC. Moreover, these images are representative of the inherent variability in terms of severity that can be found in clinical practice, from small isolated cases, to multiple deposits, to larger accumulations of fluid. These scans were manually annotated by a trained expert to accurately segment all the targeted regions of CSC-related pathological fluid. The images range in resolution from $760\times 450$ to $1536\times 500$. For compatibility with all the models, and to ensure a fair comparison, all images were resized to $512\times 512$ pixels during pre-processing. The protocols followed during the development of this project were conducted in accordance with the Declaration of Helsinki, approved by the local Ethics Committee. A representative example of these OCT images, as well as the corresponding annotation indicating the pathological region can be found in Fig. 2.

#### Methodology

In this study, different CNN architectures were trained and validated to determine the one best suited for the automatic segmentation of SRF regions in OCT images associated with the chronic CSC disease. In order to achieve this goal, two backbone segmentation architectures were trained and validated: Feature Pyramid Network (FPN) [31], and U-Net [32]. These architectures were modularly combined with three different encoder architectures, by substituting and adapting the corresponding encoder part of each architecture. With an aim to explore how models of different complexity adapt to this task, three different encoder architectures were selected for this task: MobileNet [33], DenseNet [34] and ResNet [35].

**Fig. 3:**
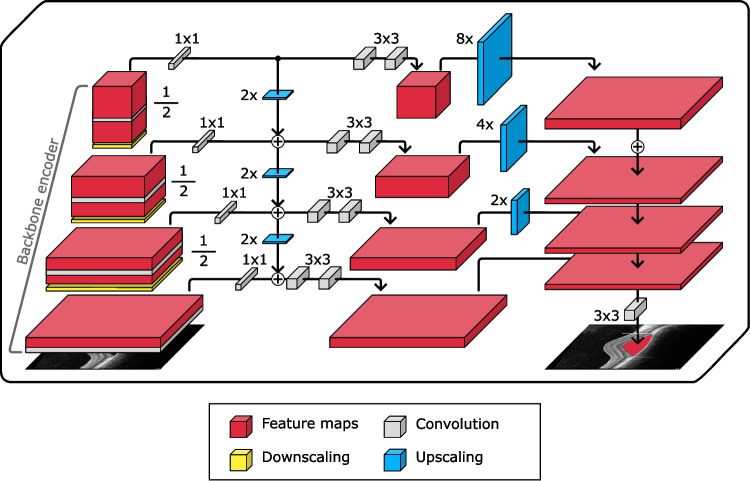
Base structure of the FPN backbone segmentation architecture. Features are extracted and progressively refined. At the later stages, the extracted features are upscaled and stacked before passing on to the segmentation head which outputs the segmentation mask

**Fig. 4:**
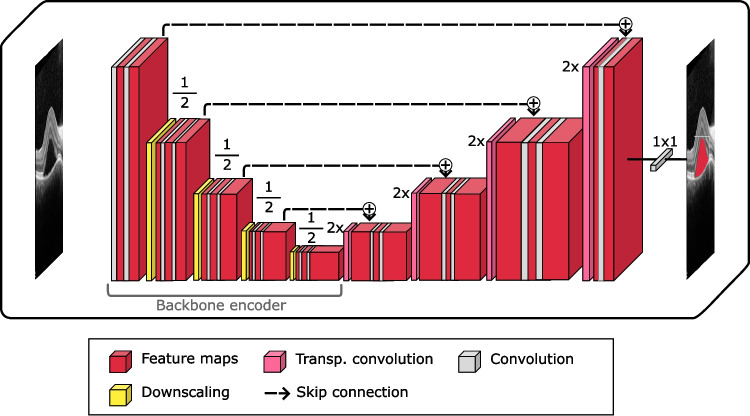
Base structure of the U-Net backbone segmentation structure. At each scale level, the extracted features are concatenated into the corresponding layers in the later part of the structure, bypassing the deeper levels

On the one hand, the FPN architecture is a convolutional, pyramid-shaped, top-down neural network with scale-invariant lateral connections originally intended for image classification [31] but later adapted for semantic segmentation [36]. This CNN architecture was specifically developed for focusing on detection at multiple scales, by merging feature maps from lower and deeper layers, and has found application in several related medical image segmentation tasks [37, 38]. This detection at different scales can be of great help to the segmentation of retinal fluid due to the different degrees of affectation that these images can present. Being able to accurately detect small buildups as well as large accumulations can improve the robustness of the models. Figure 3 displays a schematic view of this architecture. On the other hand, the U-Net architecture was specifically developed for medical image segmentation, and has been successfully applied to similar problems (for reference, [39–41]). By using skip connections between different levels of its contracting and expanding path, this architecture enables the transmission of both high- and low-level features to the final layers, enabling a more comprehensive analysis of the information contained in the images, also improving the detection of fluid buildups at different scales. A summarised view of this backbone architecture can be found in Fig. 4.

The three encoder architectures that were selected represent different levels of complexity. The MobileNet-v2 architecture [33] makes use of linearly separable and depth-wise convolutions in order to create a lightweight model. This results in a highly efficient architecture, with the smallest parameter count of those considered in this work. The DenseNet architectures [34] make use of densely connected layers in which the features are transmitted forward and concatenated along the network, using bottlenecks at the end of each dense block for limiting the explosion in the number of features. This allows these models to achieve remarkable depths in terms of layers while avoiding the vanishing gradient problem. The DenseNet-169 architecture was selected for this work as a balance between efficiency and complexity. Finally, the ResNet architectures [35] make use of residual blocks, in which features are transmitted forward via skip connections, adding them to the deeper features instead of using concatenation. These models allow for a greater complexity in terms of trainable parameters. The ResNet-34 architecture was selected for this work, representing the most complex model of those considered. For ease of comparison, a summary of the trainable parameters for each architecture configuration can be found in Table 1.

**Table 1:** Number of trainable parameters and multiply and accumulate operations for each configuration of backbone segmentation architecture and modular encoder

	FPN		
	MobileNet-v2	DenseNet-169	ResNet-34
Parameters	4 × 10^6^	15 ×10^6^	23 × 10^6^
Operations	10 × 10^9^	27 ×10^9^	27 × 10^9^

	U-Net		
	MobileNet-v2	DenseNet-169	ResNet-34
Parameters	7 × 10^6^	21 × 10^6^	24 × 10^6^
Operations	14 × 10^9^	38 × 10^9^	31 × 10^9^

The experiments that were performed were designed to allow a fair comparison between the various model configurations. To this end, a 5-fold cross-validation strategy was adopted, partitioning the data into 5 subsets. In each partition, $60\%$ of the images were used for training, $20\%$ for validation, and the remaining $20\%$ for testing, ensuring that each image appeared in the test set exactly once for each configuration. Special care was taken to confine images from the same patient to the same set, preventing data leakage and any associated biases to sharing different images from the same patient between sets. This way, the model configurations can be compared fairly among themselves.

In order to better take advantage of the limited amount of available training data, the encoder models were first initialised to a pre-training on ImageNet. Afterwards, each model was trained on its corresponding training set. At this stage, data augmentation was applied in the form of random horizontal flipping. At the end of each training epoch, the models were validated on their corresponding validation set, extracting a loss metric that was used to detect the training stage at which the models could better generalise to images not used during training. For this matter, after a fixed training length, a checkpoint of the models at the stage with the lowest validation loss was selected for testing.

The models were trained using Dice overlap loss [42] due to its performance in similar unbalanced segmentation tasks in medical imaging (for reference, [43–45]) Adam [46] was used for optimisation, with a learning rate of $1\times 10^{-3}$, $\beta _1 = 0.9$ and $beta_2 = 0.999$. These models were trained with a batch size of 16 images for a maximum of 400, which was empirically found to be sufficient for model convergence.

#### Evaluation

In order to achieve a comprehensive assessment of the performance of the segmentation models, the Accuracy, Recall, Precision, Jaccard index, and Dice coefficient metrics were employed in the evaluation of the models. Collectively, these metrics can provide a thorough validation of how these models perform.

### Intra- and Inter-expert Analysis

In order to comprehensively study the subjectivity associated to the manual segmentation of fluid regions in OCT images, as well as to thoroughly validate and assess the robustness of the trained models, an intra- and inter-expert analysis was conducted using a separate dataset consisting of both CSC and control patients. The aim of this analysis is to shed light on the differences that may arise due to expert variability, as well as to provide a benchmark against which the performance of the trained models can be compared.

#### Dataset

An independent dataset, distinct from the one that was used for the model training and validation, was employed for this analysis. This dataset was comprised of a total of 100 OCT images from different patients, 85 of which displayed signs of CSC and 15 displayed healthy eyes. These images were acquired with the Heidelberg spectralis^®^ platform, at resolutions ranging from $760\times 450$ to $1536\times 500$ pixels. As in the previous case, and for compatibility with all the models, these images were resized to a standard size of $512\times 512$ pixels during pre-processing. Two different expert annotators were separately asked to manually label the presence and location of CSC-related fluid accumulations for each OCT image. As in the previous case, the dataset was collected after approval from the local ethics committee, following the tenets of the Declaration of Helsinki. Examples of the manual labelling by the experts can be found in Fig. 2.

#### Methodology

The intra- and inter-expert analysis consists of two parts. The first part is aimed at assessing the robustness and consistency of the trained automatic segmentation models. The second part is aimed at studying the subjectivity and the differences in criteria between the human experts, as well as to validate the performance of the automatic models in this context.

In the first, intra-expert analysis, the different instances of models trained in the first stage are compared among themselves. For each architectural configuration, the five models, each one trained on a cross-validation subset, were separately used to segment this independent dataset. Then, the segmentations produced by these models were compared among themselves, extracting a measurement of how similar the segmentation results are. This, in turn, can allow the comparison of the consistency and robustness of the models when trained using different sets of images. A low variability between the results of different models of a single configuration can indicate a better robustness to training with different samples, and better performance in generalisation to unseen images.

In the second, inter-expert analysis, the segmentations produced by the two experts are compared with the models that produced the highest results for each backbone in the first stage. Three different scenarios were considered in this analysis:

1. *Comparison of manual segmentations produced by the expert annotators*: This comparison was performed to study the impact of differences in criteria when diagnosing this pathology, as well as to set a reference of the magnitude of inter-expert differences.
2. *Comparison of manual segmentations with the automated segmentations produced by the best performing models*: This comparison was conducted in order to validate the segmentation models against an external, unseen dataset, highlighting any potential biases, and comparing the performance of the automated models with inter-expert variability.
3. *Comparison of automated segmentation models*: The automatic segmentation models were also compared among themselves to identify any potential biases resulting in each configuration.

In line with previous work in the segmentation of ophthalmic imaging [47], the total area segmented as SRF was used as a uni-dimensional indicator for each expert’s segmentation. This value was calculated for each segmented image and used to create a Bland-Altman plot for each comparison. This plot offers a simple way of assessing bias between mean differences, and of estimating an agreement interval between the experts [48–50].

#### Evaluation

To provide a comprehensive summary of the intra- and inter-expert comparative analysis, the Limits of Agreement (LoA) of the Bland-Altman plots were calculated at a confidence level of $95\%$ ($\alpha =1.96$). $LoA = \overline{x}\mathop {\pm }\alpha \times sd$, where $\overline{x}$ and *sd* denote the average and standard deviation of area segmented as fluid in all images in the set, respectively. These LoA can be used to calculate the amplitude between the upper and the lower LoA as a measurement of the disagreement between the experts, with wider amplitudes showing increased disagreement. Furthermore, the mean difference between the areas segmented by each expert can be used as a measurement of any existing bias, with higher values indicating the first expert tends to over-segment relative to the second one, and smaller values indicating the opposite. Aside from calculating similarity measurements in terms of the total area segmented, the Dice coefficient, Cohen’s $\kappa$ coefficient, and the Mean Square Error (MSE) were also computed as a measurement of the specific similarity between automated and manual segmentation masks.

### Software and Hardware Resources

The experiments were performed using the Python language (v.3.8.10). The PyTorch library (v.1.12.1) [51] was used to train and validate the models, while the Segmentation Models Pytorch library (v.0.3.1) [52] was used for model configuration and pre-trained weight acquisition. Statistical calculations were done using the statsmodels (v.0.13.5) and SciPy (v.1.10.1) In terms of hardware, the models were trained and validated using an AMD EPYC 7763 64-Core CPU, with 504GB RAM and two NVIDIA A100 GPUs.

## Results and Discussion

### Automatic Segmentation of Fluid Regions

**Fig. 5:**
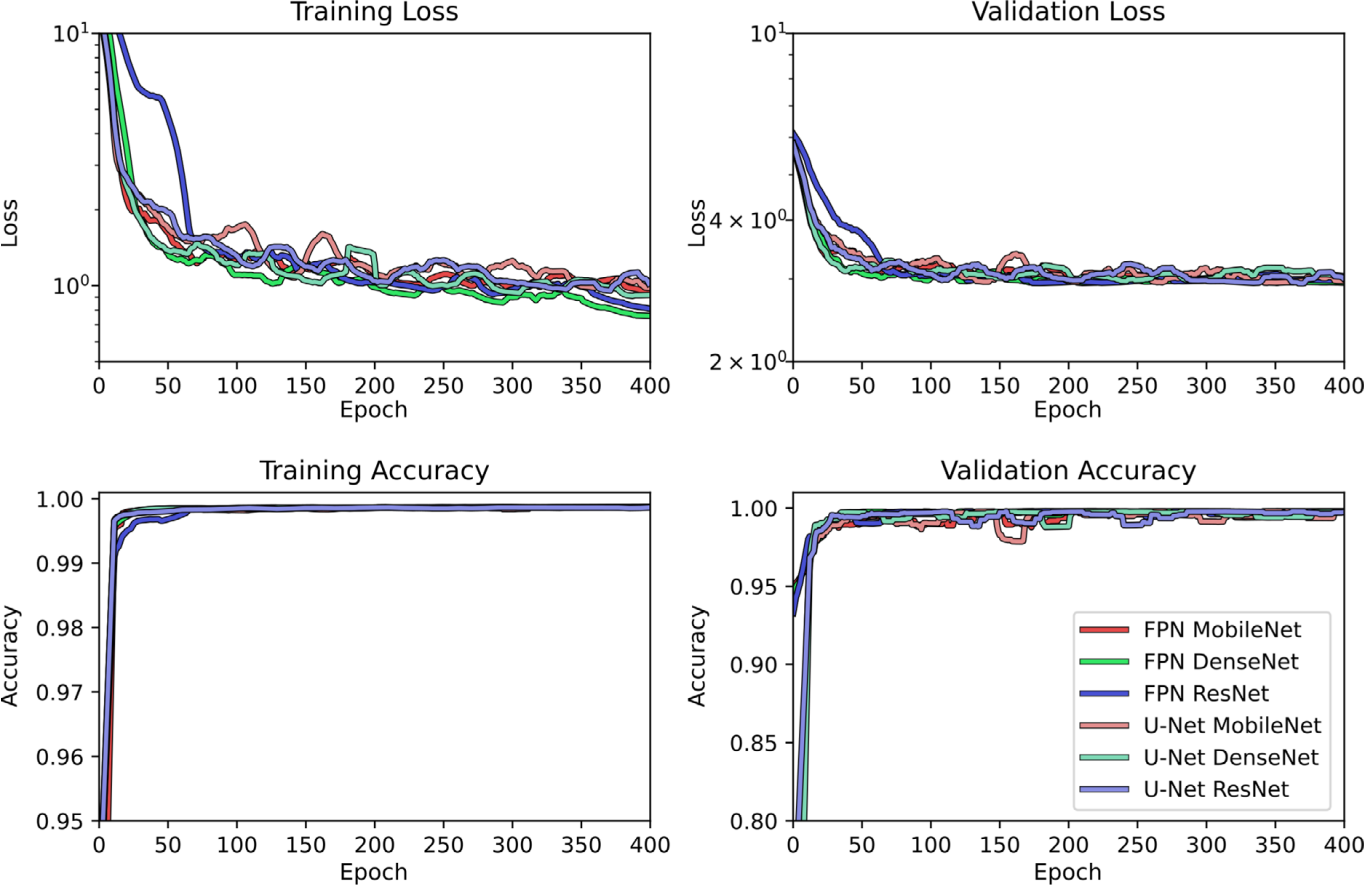
Training and validation curves describing the average training and validation loss and Accuracy for the models trained across every configuration

**Table 2:** Test results for each segmentation architecture configuration

Backbone	Encoder	Par.	Accuracy	Recall	Precision	Jaccard	Dice
FPN	MobileNet	4M	0.998 ± 0.002	0.897 ± 0.167	0.829 ± 0.035	0.752 ± 0.121	0.853 ± 0.086
	DenseNet	15M	0.998 ± 0.002	0.912 ± 0.135	0.825 ± 0.042	0.761 ± 0.101	0.861 ± 0.069
	ResNet	23M	0.998 ± 0.002	0.893 ± 0.185	0.828 ± 0.035	0.747 ± 0.136	0.849 ± 0.098
U-Net	MobileNet	7M	0.997 ± 0.002	0.871 ± 0.179	0.824 ± 0.041	0.729 ± 0.136	0.837 ± 0.095
	DenseNet	21M	0.998 ± 0.002	0.909 ± 0.149	0.827 ± 0.048	0.757 ± 0.136	0.859 ± 0.072
	ResNet	24M	**0.998 ± 0.001**	**0.918 ± 0.123**	**0.832 ± 0.051**	**0.769 ± 0.085**	**0.868 ± 0.056**

Values shown as average ± standard deviation of the models trained in the five cross-validation subsets

Par. denotes the number of parameters

Highest results shown in bold

The six model configurations were trained and tested as described in Section 2.1.2. Figure 5 displays the training and validation loss and Accuracy curves. These curves show that the models converge in validation before the maximum allowed number of epochs. The average epoch at which the models achieved the best results in terms of validation was $253\mathop {\pm }91$. Generally, all the configurations display a similar behaviour during training. The models using the MobileNet encoder architecture display the highest variability during training, and show generally higher loss than the others. The models which incorporated a DenseNet encoder architecture show generally lower training loss than the others, and seem to be able to achieve good results quickly in the early stages of training. The remaining models, which used the ResNet architecture, take longer to adapt, with higher loss in the early stages, but settling in lower values at the later stages, as can be expected from the most complex architecture in terms of trainable parameters.

After selecting the best training stage for each model in terms of the checkpoint with lowest validation loss, the models were evaluated on their corresponding test subset. The results of this test are shown in Table 2. A repeated measures ANOVA test was performed in order to verify whether there are significant differences between the results achieved by the models. Significant differences were found for the Accuracy ($p = 0.002$), Precision ($p = 0.008$) and Recall metrics ($p = 0.032$). Differences could not be considered significant for Jaccard ($p = 0.081$) and Dice ($p = 0.092$) at $\alpha = 0.05$. For the FPN backbone architecture, the best results are achieved by the DenseNet encoder architecture, with a Dice coefficient of up to $0.861\mathop {\pm }0.069$. Conversely, for the U-Net backbone architecture, the best results are obtained by the most complex model using the ResNet encoder architecture, with a Dice coefficient of up to $0.868\mathop {\pm }0.056$. Generally, more complex models seem to achieve better results, with the exception of the combination of FPN and ResNet architectures, which achieve results quite similar to the configuration using the MobileNet encoder. The models belonging to this configuration show higher losses when adapting to the task in the earlier stages.

The lack of a shared publicly available dataset aimed at the segmentation of CSC-related fluid regions precludes a fair comparison between this work and others in the literature. With this in mind, the study by Rao et al. [28] reports values of 0.936 for Precision, 0.890 for Recall, and 0.910 for Dice, using a private dataset and pre-processing stages. The previous work by de Moura et al. [29] achieved values of 0.879 for Jaccard, and a 0.965 for Dice, on a different dataset. The end-to-end configuration with the highest results considered in this work (U-Net with ResNet encoder) achieved average values of 0.832 for Precision, 0.918 for Recall, 0.769 for Jaccard and 0.868 for Dice. While these values are not directly comparable since they are measured against different datasets, they are indicative of these models achieving a performance at least competitive with those of the state of the art [28, 29]. In this scenario, an intra- and inter-expert analysis can provide valuable insight and assess the performance of the models presented in this work. An inter-expert analysis involves the comparison of the results obtained by different models within the same study, rather than by comparing the results with other works. By comparing the results of the architectural configurations within the same study, it is possible to identify the strengths and limitations of each model, as well as to accurately determine which configurations can perform better in specific scenarios. Furthermore, the addition of an intra-expert analysis can shed light on the variability and robustness of the trained models under different training scenarios, ultimately providing a more comprehensive evaluation of the models and highlighting the challenges and opportunities for further research.

### Intra- and Inter-expert Analysis

**Table 3:** Intra-expert analysis results. Metrics are extracted by comparing the segmentations produced by every model against all those produced by the other models within a configuration, then averaging across all models belonging to each configuration. For MSE, lower values are better

		Accuracy	Jaccard	Dice	$\kappa$	MSE
FPN	MobileNet	$0.999\mathop {\pm }0.001$	$0.934\mathop {\pm }0.029$	$0.965\mathop {\pm }0.015$	$0.965\mathop {\pm }0.016$	$0.016\mathop {\pm }0.005$
	DenseNet	$0.999\mathop {\pm }0.001$	$0.944\mathop {\pm }0.026$	$0.971\mathop {\pm }0.014$	$0.971\mathop {\pm }0.014$	$0.012\mathop {\pm }0.002$
	ResNet	$0.999\mathop {\pm }0.001$	$0.939\mathop {\pm }0.033$	$0.968\mathop {\pm }0.017$	$0.967\mathop {\pm }0.018$	$0.014\mathop {\pm }0.001$
U-Net	MobileNet	$0.999\mathop {\pm }0.001$	$0.975\mathop {\pm }0.030$	$0.975\mathop {\pm }0.016$	$0.972\mathop {\pm }0.016$	$0.007\mathop {\pm }0.001$
	DenseNet	$0.999\mathop {\pm }0.000$	$0.955\mathop {\pm }0.024$	$0.977\mathop {\pm }0.012$	$0.976\mathop {\pm }0.013$	$0.008\mathop {\pm }0.001$
	ResNet	$0.999\mathop {\pm }0.000$	$0.954\mathop {\pm }0.019$	$0.976\mathop {\pm }0.010$	$0.977\mathop {\pm }0.010$	$0.007\mathop {\pm }0.002$

In the intra-expert analysis, the models were evaluated by comparing the segmentation results produced by each model belonging to a configuration among themselves, using the second independent dataset. Thus, for every configuration, the output segmentation maps of every model were compared pair-wise with those of every other model. The results were then averaged across all the models belonging to said configuration. This allows the extraction of a single summary value for each metric for every architecture configuration. The results can be found in Fig. 3.

The results show that these models are highly robust, without significant deviations, even with trained with different images and using an external set of images under the same conditions. Between the two base segmentation backbone architectures that were considered, the models that used the U-Net architecture seem to display less variability among themselves than those trained with the FPN architecture, with all U-Net models achieving better results in every metric. This can be indicative that the U-Net architecture is less prone to overfitting to training data than the FPN. Models using the U-Net architecture are generally more complex in terms of trainable parameters (Table 1), which can suggest that more complex architectures may fare better in terms of variability and robustness. This fact is also supported by models using the MobileNet architecture showing greater instability than more complex ones.

**Fig. 6:**
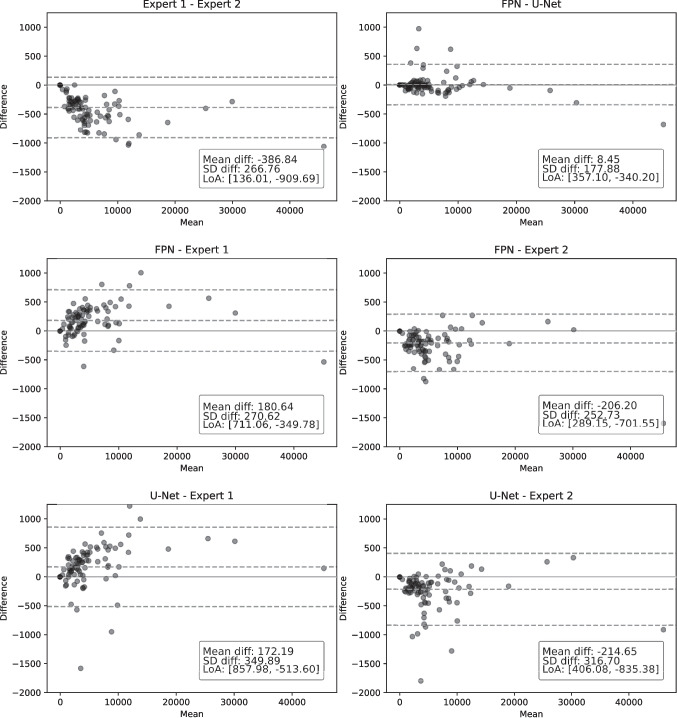
Bland-Altman plots showing the one-on-one comparison between the human experts and the automated segmentation models. The horizontal axis represents the average area segmented as CSC-related fluid in both images. The vertical axis represents the difference between the area segmented in each image in the first set and its equivalent in the second set

In the inter-expert analysis, the human experts and automated models were compared among themselves. The deep learning architecture configurations that produced the highest results were the FPN backbone with DenseNet encoder module, and the U-Net backbone with a ResNet encoder module. Within each configuration, the model with the lowest validation loss was selected to generate the segmentation masks for comparison with the human experts. Figure 6 displays the Bland-Altman plots of the three scenarios that were considered, while Table 4 shows the results from the inter-expert analysis.

**Table 4:** Inter-expert analysis results

			Amp.	MD	Dice	$\kappa$	MSE
Expert 1	-	Expert 2	1051	-387	0.952	0.951	0.229
FPN	-	Expert 1	1066	271	0.960	0.960	0.134
FPN	-	Expert 2	996	-206	0.961	0.960	0.140
U-Net	-	Expert 1	1378	172	0.957	0.956	0.132
U-Net	-	Expert 2	1248	-214	0.961	0.960	0.139
FPN	-	U-Net	697	8	0.960	0.959	0.138

Amp. denotes amplitude, as the difference between the higher and lower LoA in the Bland-Altman plot, a higher value indicates higher disagreement

MD is the mean difference between the number of pixels detected by the first and second experts, values further from 0 indicate a bias

These results show that the models correctly adapt to this task, achieving results that fall well within expert variability. In the first comparison scenario, the two human experts were compared. The results show that there is a bias towards the second expert, indicating that they tend to over-segment when compared with the first expert in terms of mean difference of area segmented. This can be seen in the corresponding Bland-Altman plot, where most of the examples tend to describe a descending trend. Comparing the two experts produces a Dice and a $\kappa$ coefficients of 0.952 and 0.951, as well as a MSE of 0.229. This comparison shows the most significant disagreement of those considered, and is representative of what can be expected from manual inspection in daily clinical practice. Regarding the second scenario, in which the models are compared with the experts, we can see that the models agree more with each of the human experts separately than these experts agree with each other. As established in the first scenario, the first expert may tend to under-segment, while the second one seems to have a tendency to over-segment. This is apparent also in the comparison with the models, where comparisons with Expert 1 yield a positive bias (towards the model segmenting more area) while comparisons with Expert 2 yield a negative bias (towards the second expert). All models achieve higher Dice and $\kappa$ coefficients, as well as a smaller MSE than the human experts. While the U-Net architecture achieved comparably better results during testing in the first stage, the FPN architecture seems to better align itself with Expert 1. Both architectures align similarly with the second expert. In the third scenario both models were compared among themselves. The corresponding Bland-Altman plot shows that the FPN model tends to over-segment in images with smaller patches of fluid, while U-Net segments more area in more affected images. Nevertheless, with a mean difference of 8, the bias between the models is substantially smaller than in any comparison involving the human experts. Overall, these models seem to provide good balance between the two human experts. Both deep learning-based configurations are able to find a consensus close to either of the human experts, without significant over- or under-segmentation, and finding better agreement among themselves and with either of the experts than either human expert with the other. This highlights the significant subjectivity in the process of manual segmentation, and shows that machine learning models can be used to find a consensus between experts and provide a robust and repeatable assessment of these images for the diagnosis of CSC.

**Fig. 7:**
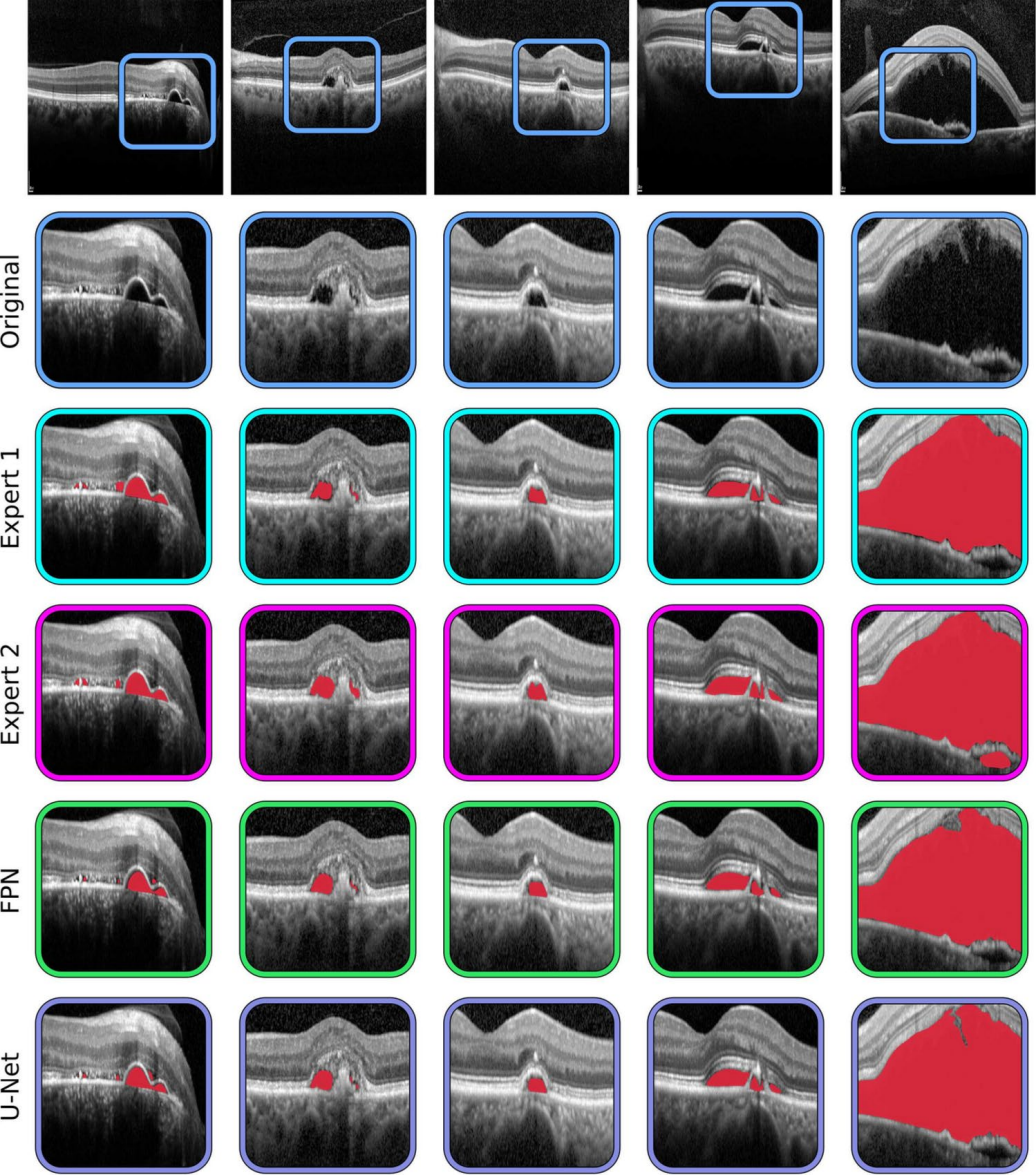
General and detailed view of the manual annotations and automatic segmentation masks of images from the inter-expert analysis performed using the second dataset. Segmentation masks overlaid with original image for ease of reference

**Fig. 8:**
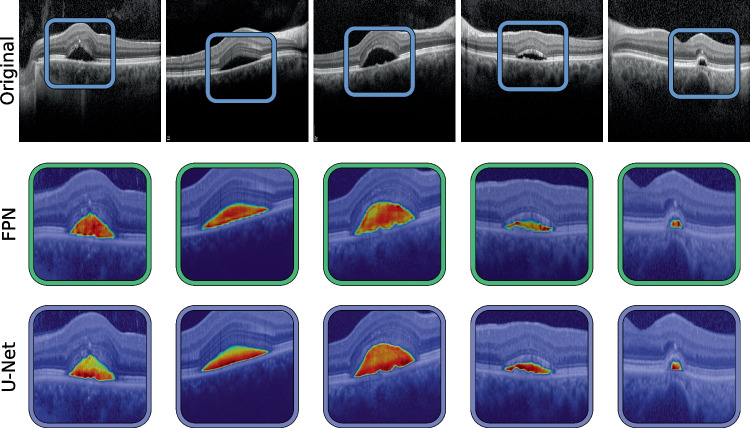
Heatmap visualisation using XGrad-CAM of the class activation for fluid in the retina generated using the best performing FPN and U-Net configurations

In order to better assess the possible differences between the manual and automatic annotations, Fig. 7 shows some visual examples of the segmentation results. This image highlights the differences in criteria regarding the boundaries of the segmented areas, as well as the size and location of smaller areas, specially those adjacent to bigger fluid accumulations. For more easily explainable results, Axiom-based Gradient-weighted Class Activation Mapping (XGrad-CAM) [53] can be used to visualise the areas that maximise network activation for the fluid detection. This way, it is possible to see the areas where the model has a higher activation and areas where the activation is lower, producing segmentation results that are easier to interpret (Fig. 8).

While this work presents many strengths, it is essential to address the following limitations. First, the images employed in this work are categorised as presenting CSC or healthy tissue, but the actual severity of the CSC images has not been graded. Second, the comparisons in this work have been limited to end-to-end deep learning architectures that have performed well in similar tasks, and other bespoke methods tailored specifically for CSC segmentation may provide better results. Finally, the methods in this work were validated using images from a single platform (Heidelberg spectralis^®^).

## Conclusions

The diagnosis of CSC is typically performed by means of an expert visually inspecting images in search for signs of the disease. This process is subjective, tiring and can lead to errors which, in turn, can translate into a late or even missed diagnosis. In this work, we have designed and validated a methodology for the fully automatic segmentation of CSC-related fluid regions in OCT images. This methodology has been implemented using a series of modular state-of-the-art segmentation architectures, representative of a spectrum of complexity. Along with a thorough comparison of all architectures, studying which are better suited for the segmentation of CSC signs in OCT images, this work is the first in the literature to propose a comprehensive intra- and inter-expert analysis to validate these models. In this study, the models are compared among themselves and with different human experts using an external validation dataset. This study can help measure the variability caused by the natural subjectivity of manual image inspection, and also provide a robust framework with which to validate deep learning models aimed at this task.

The results that were obtained show that these automatic models can perform at least at a level comparable to the experts, finding a balance between them and achieving a higher level of agreement with either of the human experts than those among themselves. The models also show a smaller bias when compared with either of the human experts, and almost none when compared with each other. These findings indicate that while differences in expert criteria may exist, deep learning models can be used to achieve a robust and repeatable segmentation of CSC-related lesions in OCT imaging, finding a consensus among experts and providing an objective and accurate segmentation of the fluid regions. These models can be used to improve the diagnosis process of the CSC, while improving patient care and prognosis thanks to an early and precise assessment of this disease.

Plans for future work involve a more detailed analysis of the different stages at which fluid may accumulate under the RPE in CSC patients. Moreover, future work could focus on including other purpose-specific architectures into the study that may provide other advantages into this task, as well as studying the data efficiency of these architectures. Finally, the inclusion of other imaging platforms to conform to a multi-expert multi-vendor dataset could help provide a more robust validation framework with which to validate future fluid segmentation methodologies.
